# Time-course evaluation of intestinal structural disorders in a porcine model of intra-abdominal hypertension by mechanical intestinal obstruction

**DOI:** 10.1371/journal.pone.0191420

**Published:** 2018-01-22

**Authors:** Ester Párraga Ros, Laura Correa-Martín, Francisco M. Sánchez-Margallo, Irma Eugenia Candanosa-Aranda, Manu L. N. G. Malbrain, Robert Wise, Rafael Latorre, Octavio López Albors, Gregorio Castellanos

**Affiliations:** 1 Department of Anatomy and Comparative Pathology, Veterinary Faculty, University of Murcia, Murcia, Spain; 2 Laparoscopy Department, Jesús Usón Minimally Invasive Surgery Centre (JUMISC), Cáceres, Spain; 3 Highlands Teaching and Research Farm (CEIEPAA), Faculty of Veterinary Medicine, National Autonomous University of México, Querétaro, México; 4 Medical and Surgical ICU and High Care Burn Unit, Ziekenhuis Netwerk Antwerpen, ZNA Stuivenberg/St-Erasmus, Antwerp, Belgium; 5 Critical Care Unit, Edendale Hospital, Pietermaritzburg, South Africa; 6 Discipline of Anaesthesiology and Critical Care Nelson R Mandela School of Medicine, University of KwaZulu-Natal, Durban, South Africa; 7 Department of General Surgery, Virgen de la Arrixaca General University Hospital, Murcia, Spain; INIA, SPAIN

## Abstract

**Background:**

A mechanical intestinal obstruction (MIO) can generate intraabdominal hypertension (IAH) that is life threatening. The intestines are very sensitive to IAH since the low splanchnic perfusion causes intestinal hypoxia, local acidosis and bacterial translocations. This may lead to acute intestinal distress syndrome (AIDS). The identification of intestinal injuries during IAH and its correlation with clinical parameters as the abdominal perfusion pressure (APP), the gastric intramucosal pH (pHi) and lactic acid (Lc) are still unknown. This study aimed to evaluate the sequence of intestinal histopathological findings in an MIO model and to analyze potential relationships with parameters currently used in clinical practice (APP, pHi and Lc).

**Material and methods:**

Twenty pigs were divided into three groups: a control group (n = 5) and two experimental groups with 20 mmHg (G1, n = 10) and 30 mmHg (G2, n = 5) of IAH by MIO. The pressures were maintained for 3 hours, except in 5 animals in G1 where it was maintained for 5 hours. The APP, pHi and LA were recorded and biopsies of the terminal ileum were taken every 30 minutes in all groups. The intestinal damage was graded according to the Park Score.

**Results:**

Intestinal injuries were found in 42.9% of pigs in the experimental groups. The lesions were independent of the level and duration of IAH. Although APP and pHi were slightly lower in injured animals (I +) of G1 and G2, there were no significant differences among those uninjured (I-). Lc was significantly increased in all I+ pigs from the onset of IAH.

**Conclusion:**

The IAH by MIO causes intestinal lesions from the first 30 minutes with concurrent decreases in APP and pHi and increases in Lc. Lc could be the best clinical parameter related to intestinal damages with a clear difference between I + and I- animals.

## Introduction

Mechanical intestinal obstruction (MIO) is a predisposing cause of intra-abdominal hypertension (IAH) [1–3], which may lead to abdominal compartment syndrome (ACS) and multiple organ dysfunction syndrome (MODS) [4, 5]. Perfusion of all abdominal organs is negatively affected by the IAH generated during MIO result of the bowel distension. This includes the perfusion-sensitive intestinal villous brush border [6–8], and so its vascular anatomical features predisposing to hypoxia [9]. Subsequent epithelial damage and local acidosis may finally lead to bacterial translocation [2, 10–12] and result in acute intestinal distress syndrome (AIDS) due to the inflammatory mediators and cytokines that are generated in the gut promoting local and remote tissue damage [8, 13, 14].

**Fig 1:**
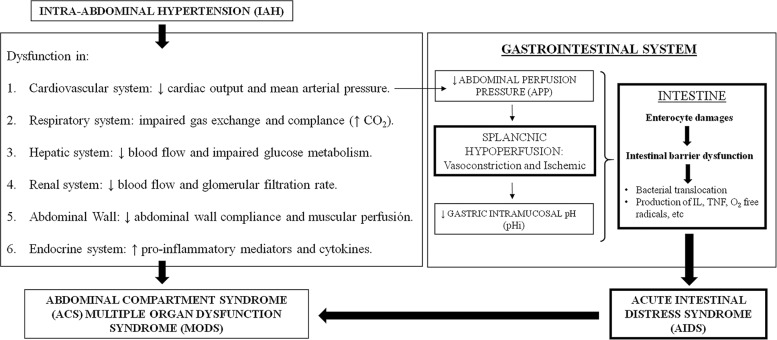
The role of the bowel in relation to intra-abdominal hypertension and abdominal compartment syndrome. Intra-abdominal hypertension (IAH) has multiple perfusion related consequences on all major organs. The decreased abdominal perfusion pressure (APP) leads to reduced splanchnic perfusion and hypoxia that promotes an anaerobic environment with a low gastric intramucosal pH (pHi). The hostile intestinal environment causes the mucosal damage, injury of the enterocytes, the intestinal barrier dysfunction and the release of inflammatory mediators and cytokines. This results in acute intestinal distress syndrome (AIDS), fuelling the development of abdominal compartment syndrome (ACS) and multiple organ dysfunction syndrome (MODS).

The identification of intestinal injury associated with IAH remains a clinical challenge, with non-specific symptoms being evident before bowel necrosis occurs [9]. Besides standard parameters of systemic perfusion, intestinal parameters of high diagnostic value such as the abdominal perfusion pressure (APP) [13, 15, 16], the gastric intramucosal pH (pHi), lactic acid (Lc) [16–20] and indocyanine green plasma disappearance rate (ICG-PDR) [21, 22] are currently used as early predictors of acidosis and intestinal ischemia (Fig 1). In recently published work [23], a porcine model of IAH by MIO was validated. The APP, pHi and Lc were three predictors for end-organ function including the bowel, but the relationship between these and the intestinal damage is poorly understood. Previous authors have observed intestinal injuries in IAH models of pneumoperitoneum [10, 24–27] and ascites [28]. But the effect of IAH on intestinal integrity as a result of MIO has not been studied before.

We aimed to study the intestine, a target organ within ACS, and the alteration in its absorption surface that may lead to an overt septic shock [13].

The goal was to evaluate in an animal model the sequence of intestinal histopathological findings during a simulation of IAH following MIO and to analyze potential relationships with parameters (APP, pHi and Lc) currently used in clinical practice.

## Materials and methods

The MIO model was previously described in a pilot validation study [23]. The project was approved by the Ethical Committee for Animal Welfare adopted by the center (reference CEBA-CCMIJU 09/25) and by the Council of Agriculture and Rural Development of the Regional Government of Extremadura.

### Animals and anesthetic protocol

Twenty Large White female pigs (23.4 ± 3.7 kg) from the animal facility of the Jesús Usón Minimally Invasive Surgery Center (CCMIJU), Cáceres, Spain, were studied. After 24 hours of fasting, the animals received intramuscular premedication with atropine, diazepam, and ketamine, followed by 1% propofol prior to intubation. Anesthesia was maintained with isofluorane, remifentanil was administered for intraoperative analgesia and 0.9% sodium chloride was used as intravenous fluid. The animals were euthanized with potassium chloride at the end of the experimental procedure and the necropsy of animals was made following guidelines of the European Animal Protection Law (Directive 2010/63/EU of the European Parliament).

### Study design

**Fig 2:**
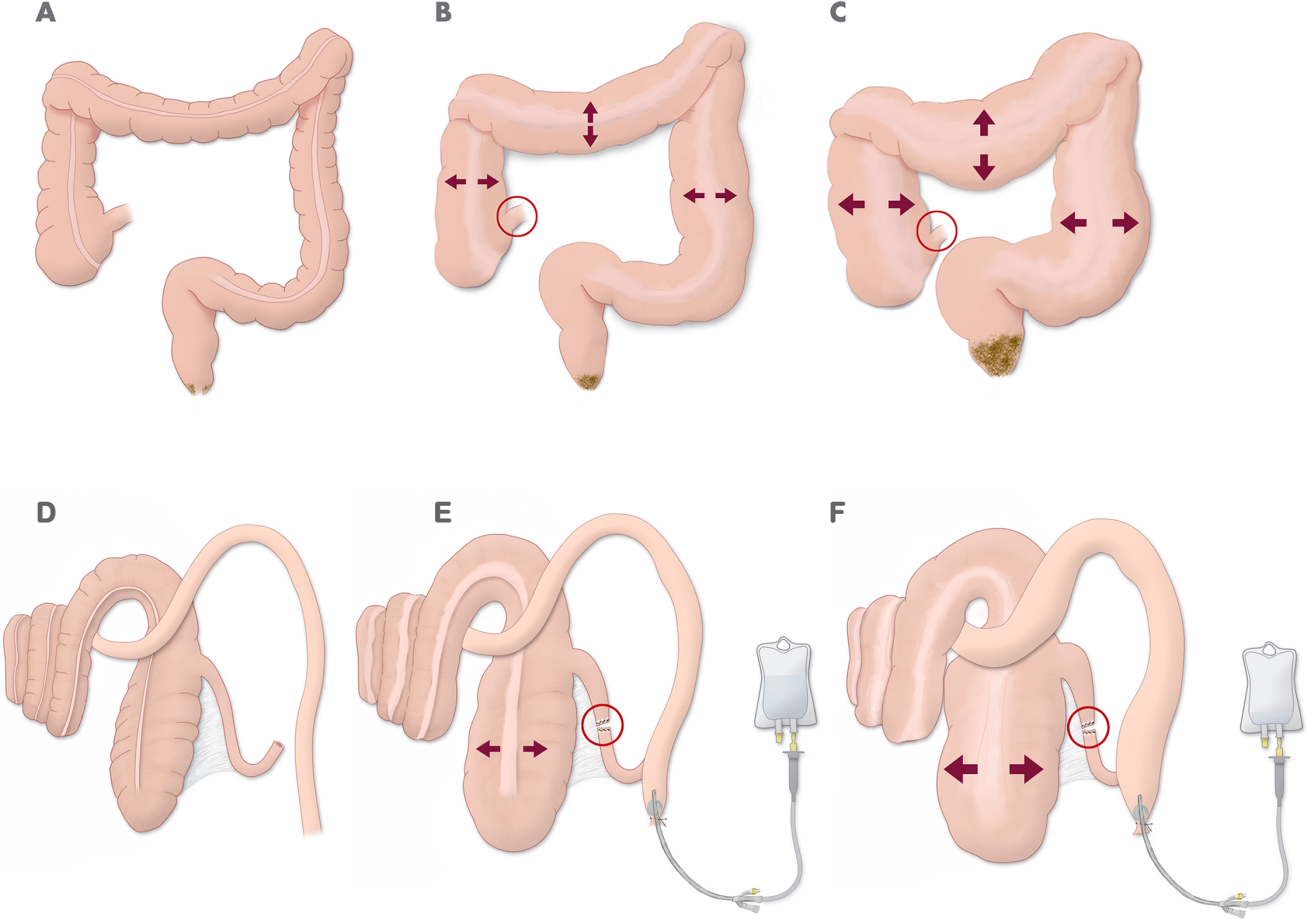
**Mechanical intestinal obstruction with competent ileocaecal valve in humans (A-C) and a porcine model (D-F).** (A) Nondistended colon (no stenosing rectal tumor). (B) Distended colon moderately with competent ileocaecal valve (red circle) and stenosing rectal tumor. (C) Colon with great distention with competent ileocaecal valve (red circle) and stenosing rectal tumor. (D) Anatomical representation of colon in a porcine model. (E) Distal ileum section (red circle) and transrectal catheterization with Foley catheter for perfusion of solution. (F) Distended colon in a “closed loop” colonic obstruction model by increased intraluminal perfusion.

**Fig 3:**
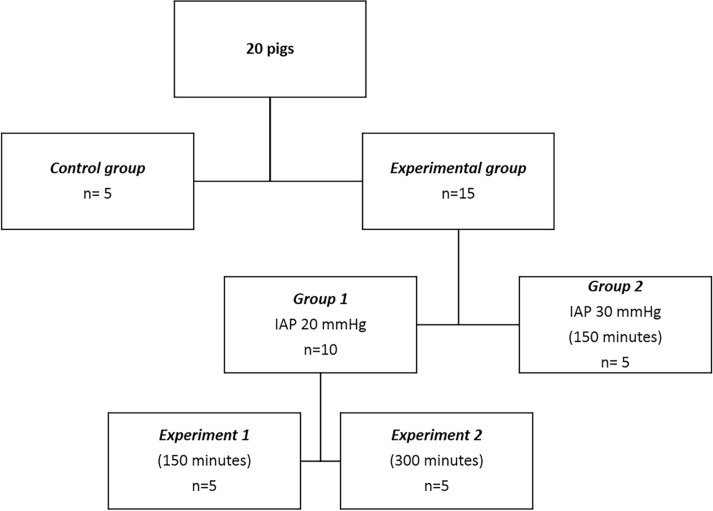
Study design. Diagram showing the different experimental groups.

The animals were divided in one control group (C, n = 5) and two experimental groups (G1 and G2) where the IAP was a consequence of experimental MIO model already established [23] (Fig 2). This model simulated the IAH through a forced laparoscopic suture at the ileocaecal valve, to achieve mechanical obstruction, and perfusion of 0.9% sodium chloride solution into the colon to increase the IAH subsequently. The pressure was maintained using a 2-way Foley catheter inserted into the rectum. In G1, an IAP of 20 mmHg was maintained for either 150 (experiment 1, n = 5) or 300 minutes (experiment 2, n = 5). In G2, an IAP up to 30 mmHg was maintained for 150 minutes (n = 5) (Fig 3). The APP, pHi and Lc were recorded in all groups every 30 minutes. Sampling times (T) were as follows: T1 (after IAP stabilization), T2 to T6 (30 to 150 minutes) and in the experiment 2 of G1 T7 to T11 (180 to 300 minutes). Biopsies of the ileum were taken for further histopathological evaluation at each of these precise sampling times. At the end of each experiment, colonic and rectal decompressions were performed. Group C had only the anesthetic procedure performed and blood and histological samples were taken at the same time intervals.

### Data collection

The IAP was measured via the direct transperitoneal route with a Jackson-Pratt catheter inserted into the abdominal cavity, and indirectly via the transvesical route with a urinary catheter connected to a Foley Manometer as previously described [29, 30]. The IAP was controlled and adjusted each time it was necessary to maintain the pressure constant in each group.

The APP was indirectly calculated from the mean arterial pressure (MAP) and IAP according to the formula: APP = MAP- IAP [15, 31]. MAP were recorded using a 5F thermistor-tipped 10 fiber optic catheter (PV2015L20N; PULSION Medical System®, Munich, Germany) placed in the abdominal aorta via the femoral artery.

The pHi was indirectly calculated according to the formula pHi = pH_a_ + LOG (P_a_CO_2_/P_g_CO_2_). The arterial pH (pH_a_) and arterial CO_2_ pressure (P_a_CO_2_) were recorded from arterial blood gas analysis on samples from the common carotid artery (i- Stat 1 Analyzer, i–Stat Cartridge EG6 + Cartridge, Abbott, USA). Continuous gastric tonometry was used to measure gastric CO_2_ pressure (P_g_CO_2_) [17, 19] (14F Tonometrics TM Catheter, Datex Ohmeda Tonometrics, Helsinki, Finland).

The Lc was used to assess the presence of anaerobic metabolism (2300 Metrolab Random Access Clinical Analyzer, Argentina).

### Tissue sampling and histological examination

**Fig 4:**
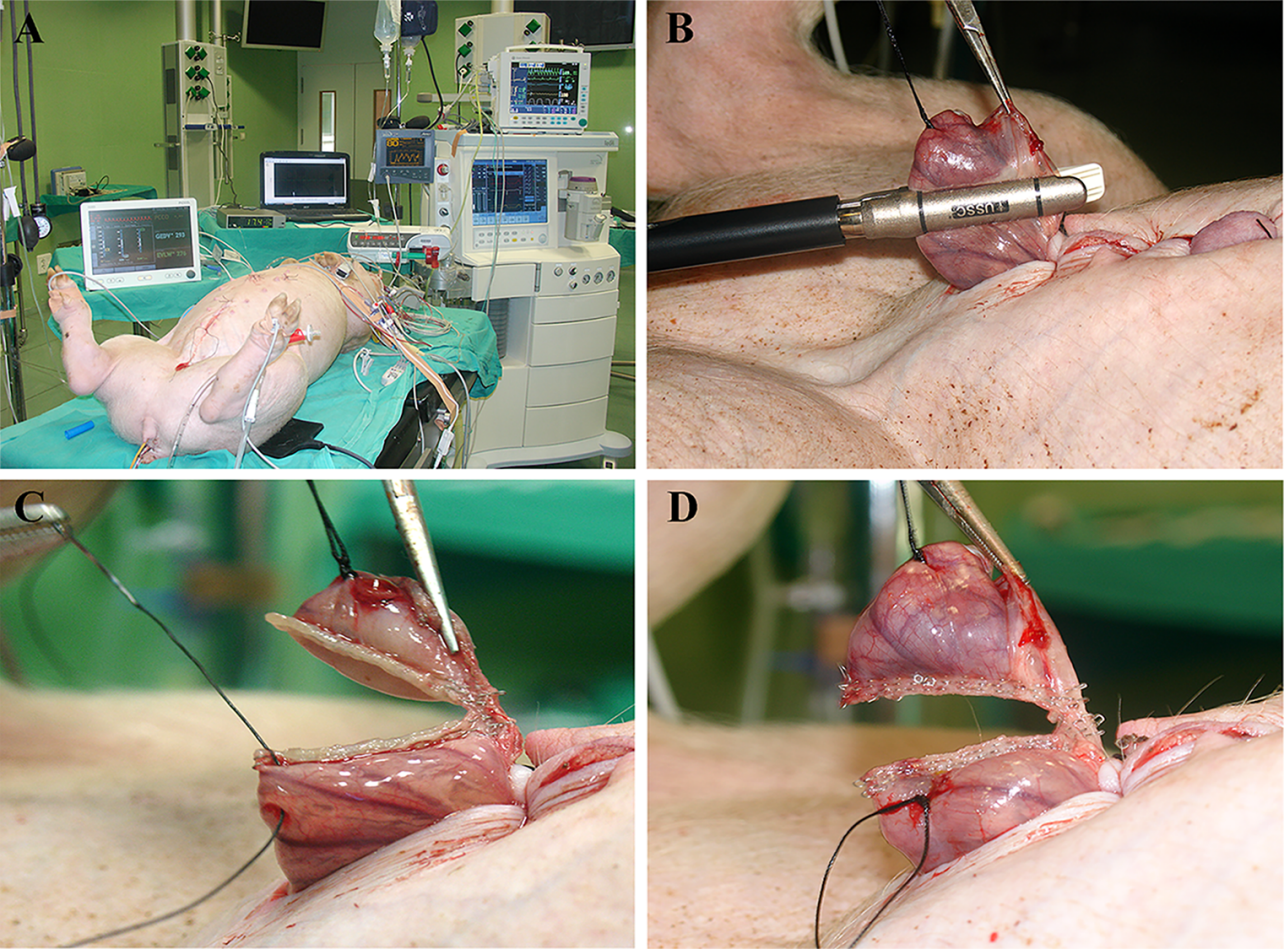
Sampling images. (A) Anaesthetized animal with all equipment connected. Note the abdominal distension due to increased intra-abdominal pressure. (B, C and D) Sequence of ileum sampling using the thread as a guide and the linear mechanical suturing device.

**Fig 5:**
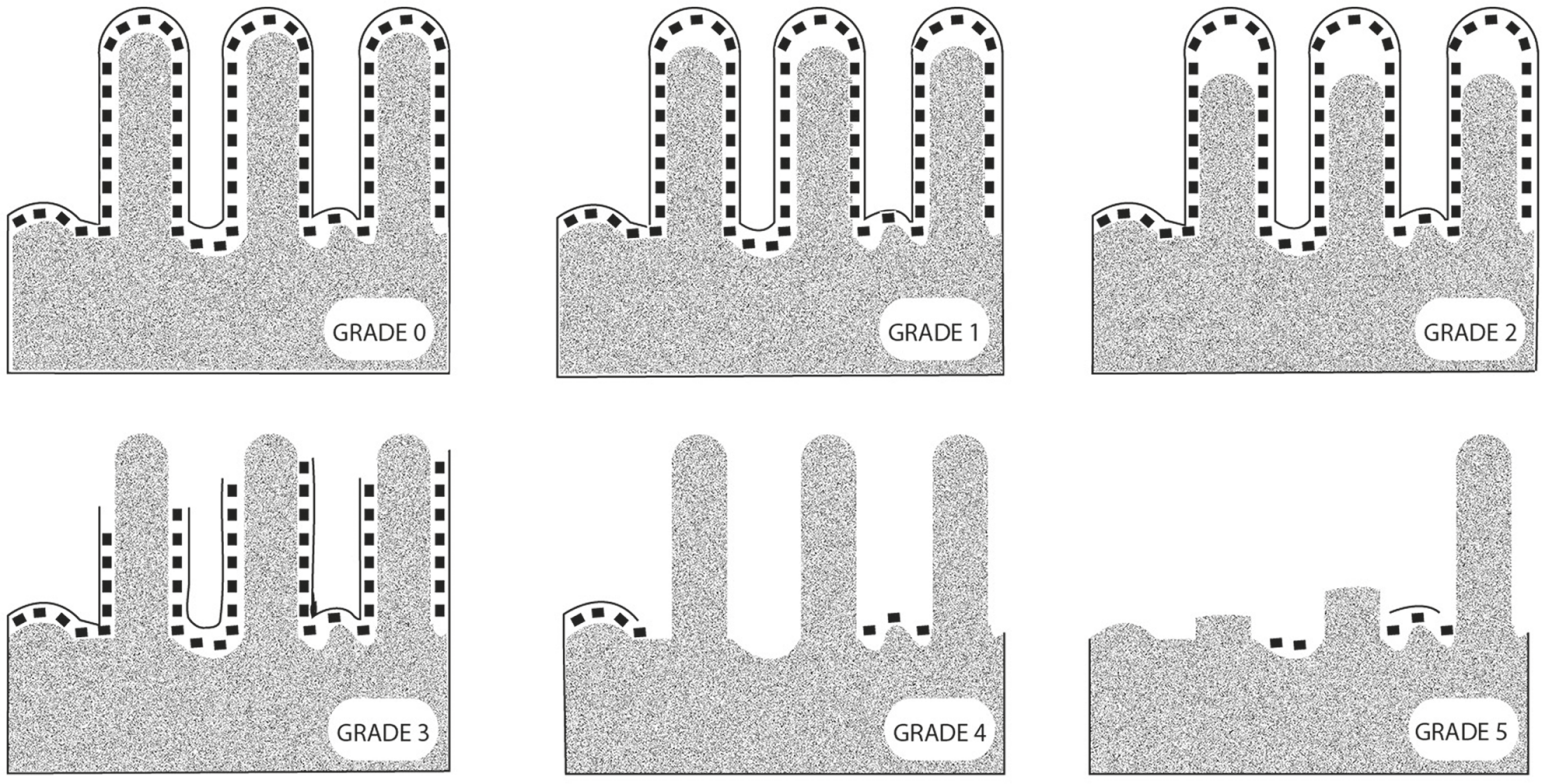
Representative images of the intestinal injury degree based on the guideline of P.O.Park [33, 34]. Normal mucosa (grade 0); subepithelial vacuolization and subepithelial space at the villi tips lower than the size of an enterocyte (grade 1); subepithelial space at the villi tips greater than the size of an enterocyte (grade 2); epithelial lifting extended along the villi sides (grade 3), denuded villi (grade 4), loss of villi (grade 5), crypt layer infarction (grade 6), transmucosal infarction (grade 7), and transmural infarction (grade 8). The grades 6, 7 and 8 were not observed in this study.

A 5 cm mini-laparotomy was performed to locate the ileocaecal valve directly. A mechanical suture was made in the ileocaecal junction, leaving a long thread as a guide to pull the ileum out of the abdominal cavity for sampling. At each sample time (intervals of 30 minutes), the ileum was exposed and a sample of 3 cm in size was taken with a linear mechanical suturing device (Fig 4). The samples were immediately fixed in a 10% buffered formaldehyde solution, routinely processed and stained with hematoxylin-eosin staining (H&E). Two entire intestinal cross sections of 5μm per sample were obtained and independently evaluated by two pathologists considering all the villi. The mucosal damage was graded according to the Park Score [32, 33] (Fig 5).

### Statistical analysis

The absence or presence of histopathological lesions of grade 3 or higher was used as criteria for allocating pigs into injured (I+) or not injured (I-) classes. The potential association between the frequencies of I+ and I-with the experimental groups G1 and G2 was analyzed with the Chi-squared test. Descriptive statistics and analysis of variance (ANOVA) with the general linear model for repeated measures were used to assess potential differences between APP, pHi and Lc. I+ and I- were used as “within-subject” factor and the group C or experimental groups as “between-subject” factors. Post-hoc differences were subjected to Bonferroni testing for p < 0.05 (SPSS, IBM Statistics Inc., Chicago, IL, USA).

## Results

### Macroscopic intestinal examination

**Fig 6:**
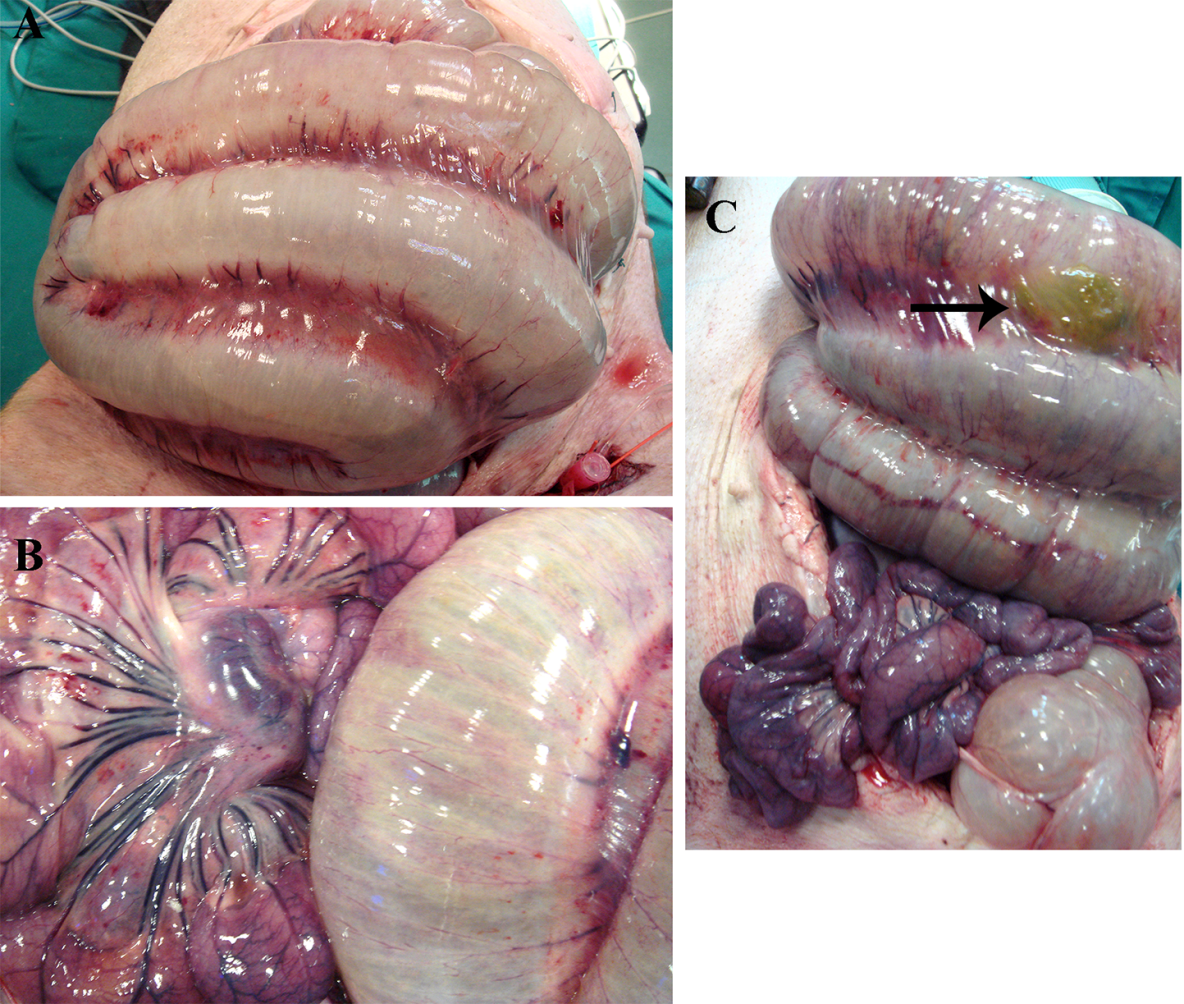
Macroscopic images of colon. (A) Dilated colon with increased intraluminal pressure. (B) Small intestine (left) and colon (right) both with edematous and congestive aspect, most remarkable in small intestine. (C) Perforation (arrow) in dilated colon at the top and congested small intestine at the bottom.

When the abdominal cavity was opened, the colon was very dilated due to the increase of intraluminal pressure (Fig 6A and 6B). The edematous, congested and thin colonic walls were observed. In fact, a colonic perforation was found in a pig (in of G1) that died and did not complete the experimental study (Fig 6C). In the small intestine, a reddish purple colouring, oedema and congestion were seen (Fig 6B). In the mesentery, some enlarged mesenteric lymph nodes were found.

### Microscopic intestinal examination

**Fig 7:**
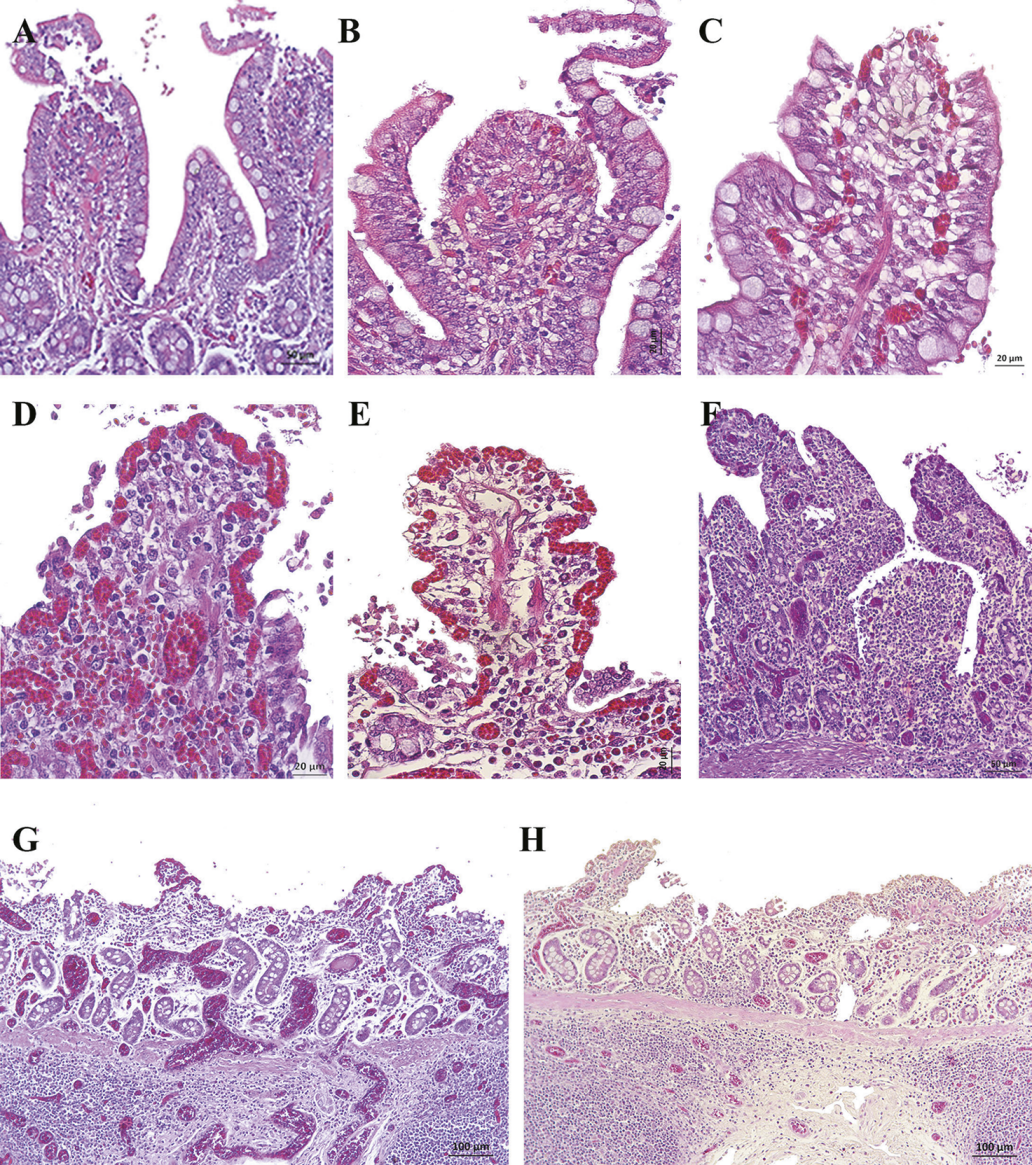
Histopathological images of ileum samples in experimental groups using the Park’s Score (H&E, haematoxyline-eosin staining). (A) Villi with large subepithelial spaces that have already broken through the epithelium at the tips (Grade 3). There were remnants of epithelial cells in the intestinal lumen. (B and C) Villi with loss of epithelium at the tips and the one that remained on the sides of the villus was detached (Grade 3). Epithelial cells appeared in the intestinal lumen. (D) Villi tips and part of the sides were denuded (Grade 3). Note the congestion and detached epithelial cells. (E) Villi and part of the crypts completely denuded (Grade 4). Note the shortening of the villi, the remains of epithelial cells in the lumen and moderate congestion. (F) Villi and crypts completely denuded. Note the moderate congestion. (G and H) Mucosal intestinal areas without villi (Grade 5). Those villi that still remained on the mucosa were denuded and very short. Note the severe congestion and lymphocytic infiltrate in mucosa and submucosa.

**Fig 8:**
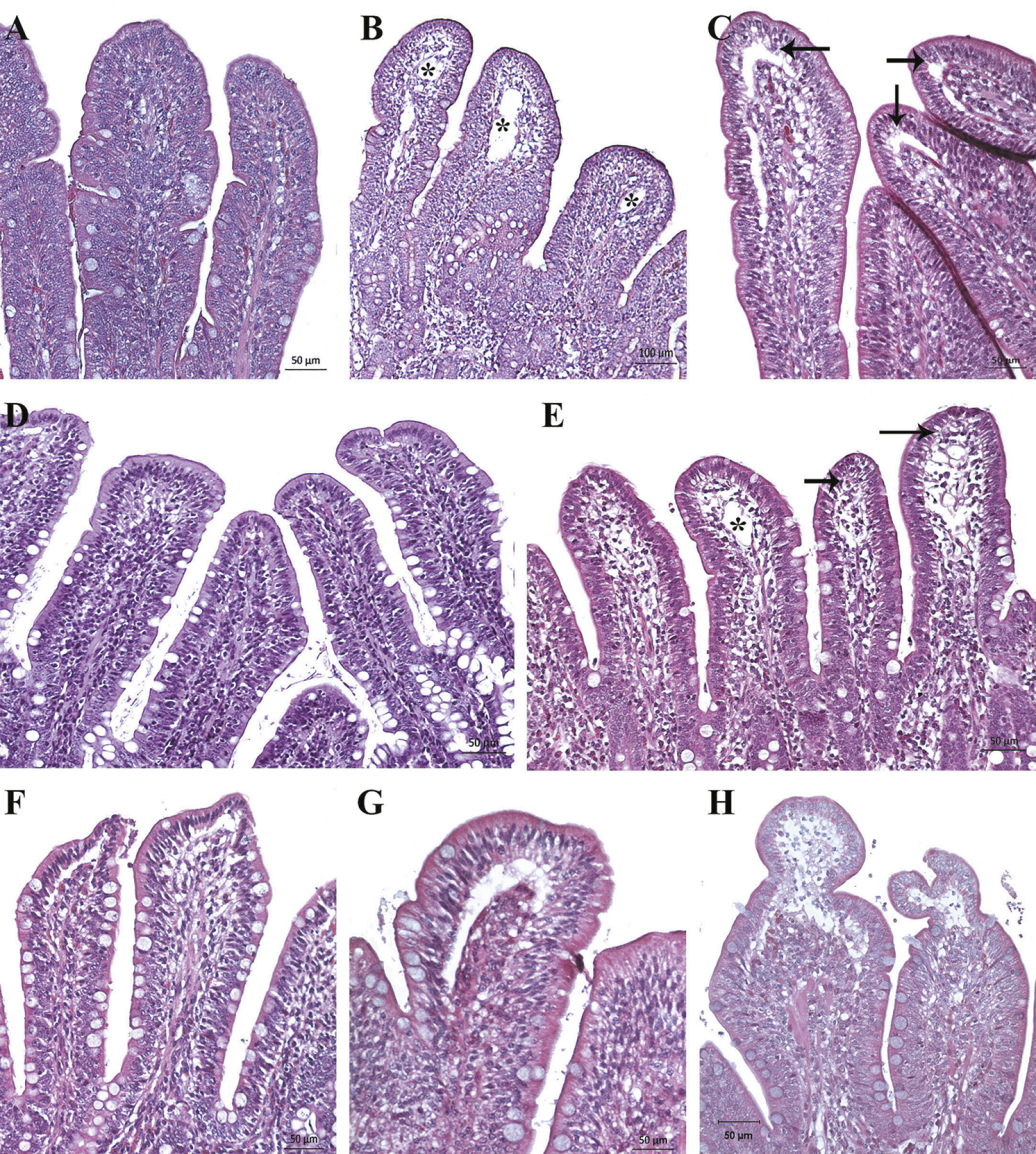
**Histopathological images of ileum samples (H&E, haematoxyline-eosin staining) in control (A-B) and experimental groups (C-H).** (A and B) Villi with intact epithelium (Grade 0) and dilated lymphatic vessel at the tips of some villi (asterisk). (C) Small subepithelial space in some villi (Grade 1, arrow). (D) Villi with preserved epithelium (Grade 0). (E) Dilation in the lymphatic vessel (asterisk) and slight subepithelial vacuolization in some villi (arrow) without forming subepithelial space (Grade 1). (F and G) Villi with small subepithelial spaces (Grade 1). (H) Villi with large subepithelial spaces that exceed twice the enterocyte size (Grade 2).

According to Park’s score classification, grades 0 to 5 were observed (Fig 5). We found that intestinal damage was not directly associated with the experimental conditions (Table 1), so different lesional severity were observed to be independent of the IAP level and the duration of the experiment (χ^2^ de Pearson > 0’05). Four of nine animals (44.4%) in G1 and three of five animals (40%) in G2 showed intestinal lesions, which the lesional degree increased progressively. When the intestine was moderately damaged (grade 3) within 2 hours of IAH, the intestine never recovered but followed a sequence of progressive damage (Fig 7). Of the nine pigs in G1, only four of them followed a clear sequence of progressive intestinal damage. Three of them (from experiment 1) reached I+ stage at T2-T3, while only one pig in experiment 2 reached it at T5. In contrast, the remaining five pigs of G1 showed minimal intestinal damage (Fig 8). In G2, two out of five pigs suffered the same sequence of structural damage from T2. In group C only slight or no damage was the common finding (Fig 8).

**Table 1:** Histopathological evaluation of intestinal samples from all animals of the study using the Park’s Score.

HISTOPATHOLOGICAL EVALUATION												
GROUP	ANIMAL	T1	T2	T3	T4	T5	T6	T7	T8	T9	T10	T11
C	P-322	1	0	1	0	1	0					
C	P-324	1	1	*	*	0	*					
C	P-326	0	0	0	0	0	1					
C	P-327	0	0	0	0	0	1					
C	P-328	0	0	*	1	*	*					
G1(Experiment 1)	P-671	2	3	3	3	4	4					
G1(Experiment 1)	P-672	0	0	1	1	1	1					
G1(Experiment 1)	P-674	1	1	1	1	1	1					
G1(Experiment 1)	P-675	1	1	4	3	4	3					
G1(Experiment 1)	P-779	1	3	4	4	4	4					
G1(Experiment 2)	P-1207	2	1	1	1	0	1	1	1	1	1	1
G1(Experiment 2)	P-1213	1	1	1	1	3	3	3	4	5	4	5
G1(Experiment 2)	P-1248	1	1	1	1	1	1	1	1	1	1	1
G1(Experiment 2)	P-1252	1	1	1	1	1	1	1	1	1	1	1
G2	P-233	2	3	3	4	4	4					
G2	P-235	1	1	0	0	1	0					
G2	P-236	1	1	0	1	1	3					
G2	P-237	1	1	1	1	1	*					
G2	P-239	2	3	3	4	4	5					

T: sampling time from the stabilization of intra-abdominal pressure (T1) to 300 minutes (T11) each 30 minutes.

* Indicates failure sample processing.

**Fig 9:**
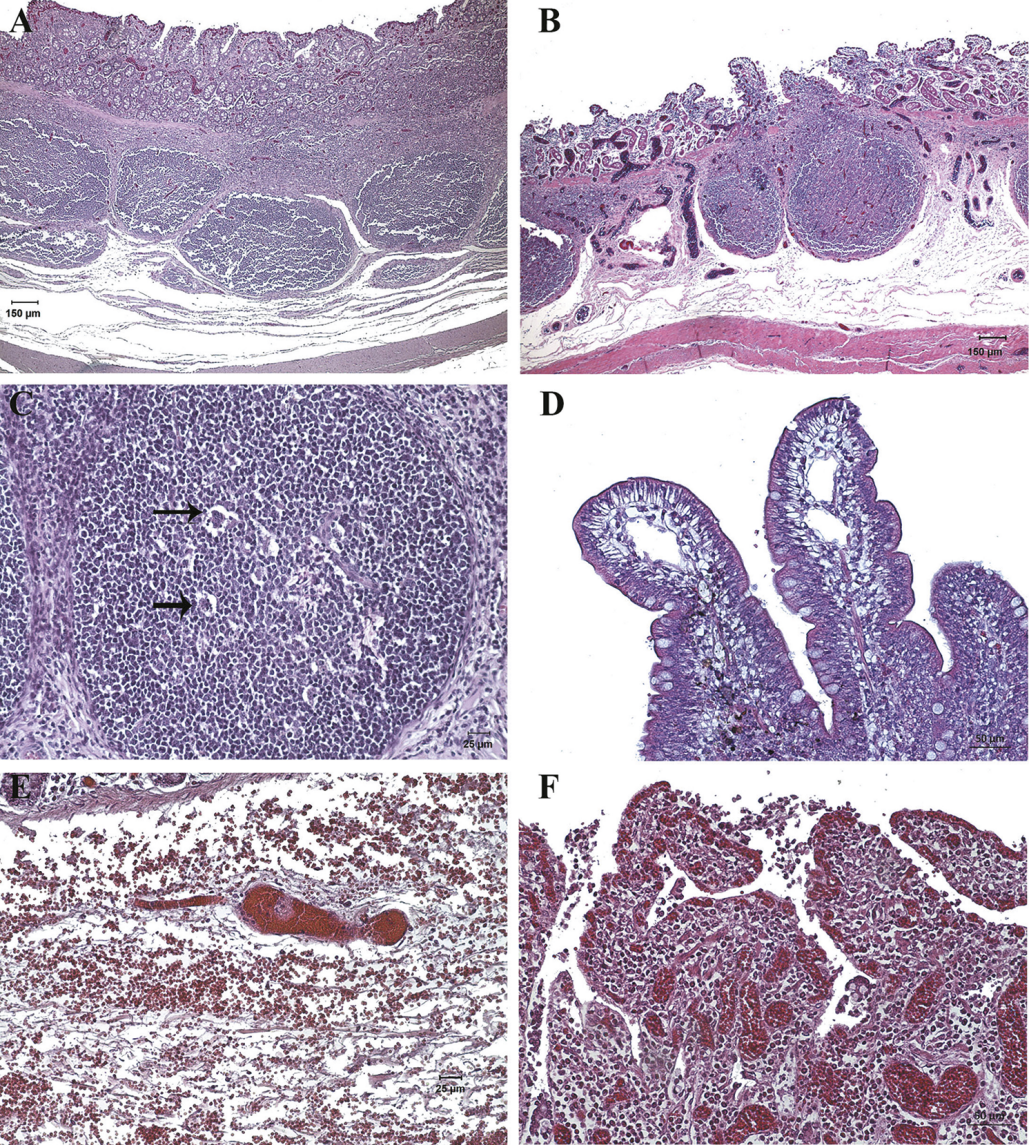
Histopathological images of ileum samples of experimental groups (H&E, haematoxyline-eosin staining). (A and B) Moderate oedema in submucosa, oedematous Peyer’s patches and severe congestion in mucosa and submucosa was observed in I+ animals. (C) Peyer’s patch with lymphocytic apoptotic forms (arrow). (D) Dilated lymphatic vessels at the villi tips in I- animals. (E) Severe congestion with formation of thrombus and moderate submucosal hemorrhage in I+ animals. (F) Villi without epithelium, with severe congestion and lymphocytic infiltrate in lamina propria observed in I+ animals.

In addition to the main lesions found in the mucosa, some interesting histopathological findings were observed. In all the samples of experimental groups, a moderate to severe submucosal and serosa oedema were found. Lymph nodes (Peyer’s patches) were slightly oedematous and apoptotic lymphocytes cells were observed occasionally. Nearly all the samples had a slight lymphocytic infiltrate in the lamina propria and lymphatic vessel dilation at the villi tips. The congestion was slight in I- animals while it was moderate to severe in I+ animals, with occasional thrombus formation and sporadic mild to moderate submucosal hemorrhages (Fig 9). In group C dilated lymphatic vessels of the villi tips with mild to moderate congestion were also occasionally observed (Fig 8).

### Clinical parameters related to intestinal injury

**Fig 10:**
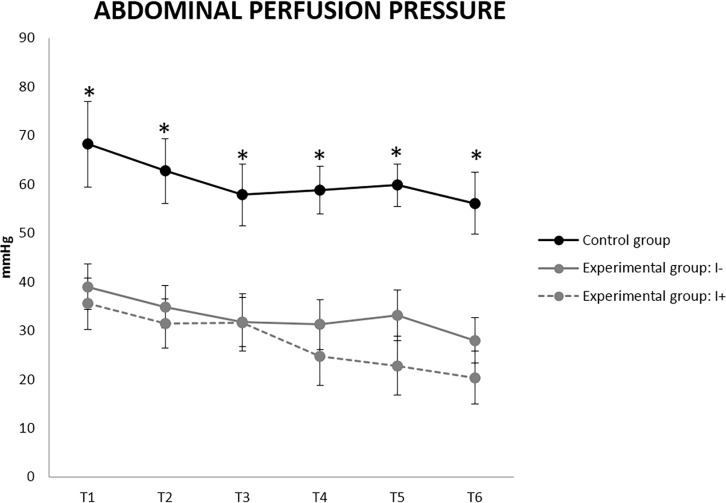
Abdominal perfusion pressure (APP) values in uninjured (I-) and injured (I+) animals (mean ± SEM). * Indicates significant differences between group C and experimental groups (p< 0.05).

**Fig 11:**
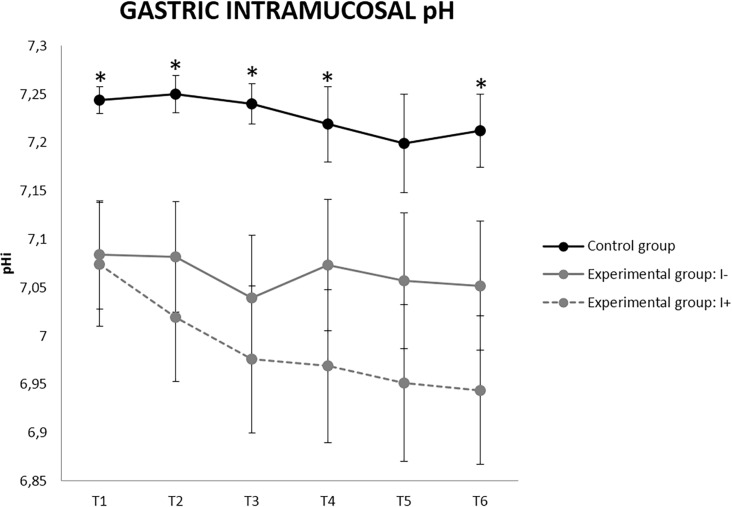
Gastric intramucosal pH (pHi) values in uninjured (I-) and injured (I+) animals (mean ± SEM). * Indicates significant differences between group C and experimental group (p<0.05).

**Fig 12:**
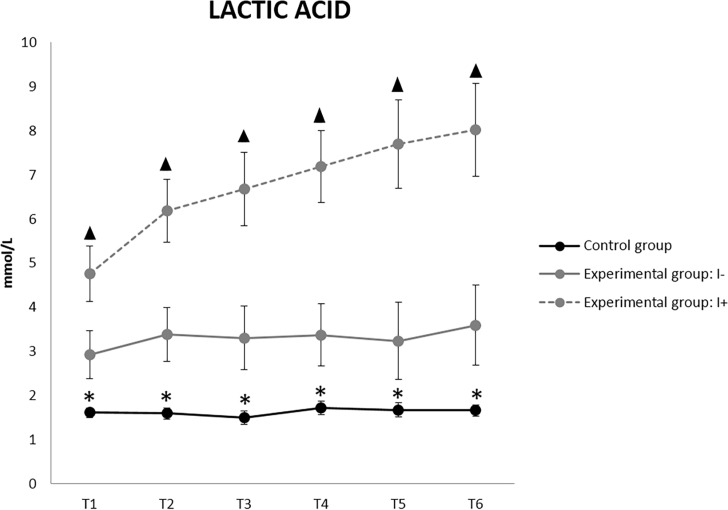
Lactic acid (Lc) values in uninjured (I-) and injured (I+) animals (mean ± SEM). * Indicates significant differences between C group and experimental group. ▲ Indicates significant differences between I+ and I- animals (p<0.05).

The clinical parameters (APP, pHi and Lc) experienced changes in all the groups. The results from T7 to T11 (Experiment 2 in G1) showed the same tendency as the first hours (Experiment 1 in G1), and as there was only one I+ pig thus preventing statistical analysis of experiment 2, the Figs 10–12 only represent the first 2.5 hours of study. The APP was significantly decreased in the experimental groups from T1 when compared to group C. While no significant differences in APP were found between I+ and I-, it is worth noting that from T4 the average APP values in I+ animals were always lower than 25 mmHg (Fig 10). This value even reached 20 mmHg at T6, and in the unique I+ pig of experiment 2 even dropped to 5 mmHg at T11. In contrast, I- animals always maintained APP values between 25 and 30 mmHg from T5 to T11.

Regarding the pHi, the experimental groups showed lower values than group C. The I+ pigs reached pHi values below 7 from T3 with a downward trend until 6.94 at T6 (Fig 11). From T7 onwards, the pHi was kept between 6.97 and 7.04 from T7 to T11 in the only I+ pig in experiment 2. In contrast, a pHi slightly higher than 7 was observed in all the I- animals, with mean values between 7.05 and 7.1.

Lc was the clinical parameter most strongly affected in the I+ pigs (Fig 12). Significant increases between the experimental and the group C, and between I+ and I- from T1 were found. Values greater than 4 mmol/L were observed from T1 and up to 8 mmol/L at T6 in I+ animals. Lc reached values of 12 mmol/L at T11 in the only I + pig in experiment 2. I- animals always had Lc levels below 4 mmol / L.

## Discussion

The sequenced design of this porcine model of IAH from MIO [23] made it possible to correlate the intestinal lesions found with the evolution of clinical parameters used in the hospitals. Despite the fact that the intestine is a perfusion-sensitive organ susceptible to IAH [6–8], some animals maintained intestinal integrity.

Studies have shown a structural intestinal damage that seemed to depend on the animal species (rats or pigs) and the IAH model used (ascites or CO_2_-pneumoperitoneum, usually). A pneumoperitoneum at 20 mmHg in rats [10, 34] caused partial denuding of the villus tips at 1 hour, which was complete in most of the epithelial surface at 4 hours [10, 26]. At the same pressure, no significant intestinal lesions were found in a porcine model after 5 hours [16], and the completely denuded intestine was only observed at 30 mmHg after 3.5 hours [16, 24, 25, 35]. However, relevant intestinal lesions were observed in our MIO model [23] at 30 minutes with 20 mmHg, where time was not decisive for the emergence of injuries provided that epithelium was intact for the first 2 hours. Also, we found the completely denuded intestine at 30 mmHg but just 1.5 hours of IAH. Compared to pneumoperitoneum or ascites models (where no intestinal damage was noted with these IAPs [36]), the IAH by MIO seems to have a more severe impact on the intestine.

These differences could be related to the method to increase IAP. In MIO model the IAH was due to a local intra-luminal effect while in pneumoperitoneum models it was by an extra-luminal effect. The gas insufflation and dispersion in the abdominal cavity is homogeneous in pneumoperitoneum models, while in MIO model there is a local effect related to the competent ileocaecal valve [37, 38] that retains the feces and enlarges the colon. However, if the ileocaecal valve is incompetent, backflow will occur towards the small intestine, increasing the size of both small and large intestines. The role of this valve is vital at the onset of IAH.

Studies have attempted to clarify the intestinal pathophysiology caused by IAH as part of the ACS process [13]. In these studies [10, 24–26, 28, 34–36, 39] the histopathological samples were taken at the end of the experiment after sacrificing the animal and releasing the IAP, then the intestinal changes during the IAH are still unknown. In our study, with the sequenced sampling method during IAH, was established that the important intestinal damage occurred between 30 and 60 minutes after the establishment of IAH. This may indicate a key moment in preventing further injury, always considering a slight individual variability.

Furthermore, our experimental design made it possible to relate intestinal histopathologic findings with APP and pHi declines as well as subsequent increases in Lc. This could guide the clinician concerning the presence or absence of intestinal integrity. In previous MIO study [23], we noted the APP and the pHi were good early predictors for low splanchnic perfusion [20, 36, 40] like other IAH studies [16, 17, 41]. In the same way, significant decreases in APP have been observed with pneumoperitoneum [16, 35] and ascites models [20, 42]. Although the APP seems to be a good indicator of low splanchnic perfusion regardless of the experimental model used, cannot be directly related to the development of intestinal lesions. We observed no significant differences in APP between I+ and I- animals even though the I+ animals had slightly lower values. As other authors have suggested [16], these findings may be related to baseline MAP values and filling status of each individual as well as by direct effects on the local vasculature. Secondly, the pHi seems to be influenced by the IAH model as well as the IAP level attained. Pneumoperitoneum models did not obtain significant pHi changes with 20 mmHg; however, significant decreases were noted after 30 minutes at 30 mmHg [16]. In ascites [20, 43] and MIO [23] models, significant declines in the pHi were observed with 20 mmHg after 2 and 3 hours of IAH, respectively. In the same way of APP, in our study no significant differences in pHi were found between I+ and I- animals even though lower values was observed in the I+ (pHi< 7). The pHi is also a good indicator of intestinal flow but its correlation with intestinal injury at early IAH stages remains unclear. What seems obvious is that ascites and MIO models cause greater splanchnic vasoconstriction compared to pneumoperitoneum resulting in increased gastric luminal CO_2_ pressure.

The Lc is other biochemical parameter used to evaluate anaerobic metabolism [28, 44, 45] and a high amount has been found intraperitoneally in a porcine model of IAH [40]. Several authors have observed subtle Lc increased (<3 mmol / L) pneumoperitoneum and ascites models [16, 20, 36, 43] of 20 mmHg with significant differences after 3 hours [20]. However, when IAP reached 30 mmHg in pneumoperitoneum, the Lc increased significantly from 3 hours with values between 3 and 10 mmol / L [16]. Others studies showed changes occurring long afterward with values between 2.5 and 8.6 mmol / L [24, 25]. In our work, the Lc was the only parameter that showed significant differences between I+ and I- animals since the beginning of the IAH. These early Lc variations may be indicative of important lesions compared to pneumoperitoneum as a result of cellular (such as the breakdown of intercellular junctions) [10] and biochemical changes induced by damage to enterocytes and the low perfusion [45]. In contrast to the APP and pHi, the Lc could be the best biochemical parameter related to intestinal damage.

In future studies, other metabolic biomarkers should be considered in relation to intestinal lesions: the cytokines (IL-6, TNFα), citrullin, liver-type fatty acid-binding protein (L-FABP) and ICG-PDR could be very useful to relate with LA values and hypoperfusion. Also, MAP values have to be considered carefully since in both experimental models and clinical practice APP is mathematically coupled to MAP.

In conclusion, IAH from MIO causes intestinal lesions in about 40% of the animals which remained and worsened when the partial or complete loss of epithelium occurs during the first hour. Lc values of 4 mmol / L or above in the first hour of IAH combined with low APP or pHi may be indicative of a critical intestinal injury. Lc is the best clinical parameter associated with intestinal lesions.
